# From empirical to analytical: Soft-tissue tension gauging in total knee arthroplasty

**DOI:** 10.1186/s43019-025-00287-0

**Published:** 2025-09-30

**Authors:** Xiang-Dong Wu, Yunfeng Zhang, Zhuyi Ma, Qi Wang, Hongyi Shao, Dejin Yang, Yixin Zhou

**Affiliations:** 1 Department of Orthopaedic Surgery, Beijing Jishuitan Hospital, Capital Medical University, Fourth Clinical College of Peking University, National Center for Orthopaedics, No. 31 Xinjiekou East Street, Xicheng District, Beijing, 100035 China; 2 Beijing Research Institute of Traumatology and Orthopaedics, Beijing Jishuitan Hospital, Capital Medical University, Fourth Clinical College of Peking University, National Center for Orthopaedics, Beijing, 100035 China

**Keywords:** Total knee arthroplasty, Soft-tissue balancing, Soft-tissue tension, Tensor, Sensor

## Abstract

Soft-tissue balancing is essential for achieving optimal outcomes in total knee arthroplasty (TKA), significantly impacting postoperative joint function, patient satisfaction, and implant longevity. Despite advancements in surgical techniques, traditional methods for evaluating soft-tissue tension remain largely subjective, leading to inconsistent outcomes and patient dissatisfaction. Recent technological developments, particularly the integration of digital devices, have shown promise in transforming soft-tissue balancing from a subjective art into a reproducible science. This manuscript is a narrative review that systematically summarizes the historical and technological evolution of soft-tissue tension gauging methods in TKA, encompassing experiential methods, mechanical tensors, and contemporary digital sensors. We critically discuss the strengths, limitations, and available clinical evidence for each method. Furthermore, this review highlights the integration of robotic systems and provides insights into future translational strategies, emphasizing artificial-intelligence-driven personalized soft-tissue balancing as a promising therapeutic direction. This review further comprehensively discusses soft-tissue tension gauging methods in TKA, providing a clear understanding of their evolution from subjective assessments to objective digital technologies. This study provides a robust theoretical foundation for the clinical application of digital tensors and robotic technologies. Integrating these technologies with artificial intelligence can effectively transform soft-tissue balancing strategies, thereby enhancing surgical precision, patient satisfaction, and clinical outcomes in TKA.

## Introduction

Total knee arthroplasty (TKA) was in its infancy, and pioneers, such as John N. Insall, have made significant contributions to the advancement of orthopedic surgery and TKA [1]. Initially, implant design was believed to be the major decisive factor influencing the range of motion (ROM) and component loosening [2]. Subsequently, Insall recognized that meticulous surgical techniques, particularly implant positioning, lower limb alignment, and soft-tissue balancing, are crucial for achieving durable TKA [3]. He further developed surgical techniques for soft-tissue release to correct fixed angular deformities and to establish balanced flexion and extension gaps [3].

Five decades later, TKA was acknowledged as one of the most successful surgical procedures for treating end-stage knee osteoarthritis [4]. However, a significant minority (approximately 10–20%) of TKA patients remain dissatisfied, often due to issues related to soft-tissue imbalance [5]. Soft-tissue imbalance, which manifests as joint instability, joint stiffness, or malalignment, is believed to cause up to one third of early TKA revisions, underscoring the critical importance of proper ligamentous balance in TKA [6]. Soft-tissue balancing relies on the manipulation of the soft-tissue envelope and ligaments, and the balancing and tensioning of the soft-tissue sleeve determine the joint gap throughout the ROM of the knee, from full extension to deep flexion [7]. Currently, there is no agreed-upon consensus on the definition of soft-tissue balance after TKA, primarily owing to the lack of intraoperative quantitative measurements of soft-tissue balance status.

Historically, soft-tissue balancing in TKA has relied heavily on the subjective judgment and tactile experience of the surgeon, and making objective, intraoperative quantification of soft-tissue tension to achieve soft-tissue balancing has long been a complex and unresolved challenge [5, 8]. Over the past few decades, increasingly sophisticated tensioning devices have been designed for intraoperative assessment or measurement of soft-tissue tension, progressing from mechanical tensors measuring gap distances and angular deviations to digital sensors embedded in trial components and, most recently, robot-compatible tensioners with real-time analytics. Despite these advancements, a comprehensive review summarizing the historical evolution and clinical implications of these soft-tissue tension gauging methods is lacking.

Therefore, this narrative review aims to systematically summarize and critically evaluate the historical and technological developments in soft-tissue tension assessment methods in TKA, highlighting their clinical relevance and outlining future directions. We provide a comprehensive overview of soft-tissue tension gauging methods in TKA, from empirical (experience-based) approaches to advanced analytical, data-driven technologies. We further categorize their development into three distinct stages—the experiential stage, the mechanical tensor stage, and the digital tensor stage (further divided into passive and active subtypes)—and systematically describe representative tools within each stage. Additionally, we discuss and compare the efficacy of these approaches, incorporating evidence from recent clinical studies. Finally, we outline emerging trends and future directions, emphasizing the integration of digital tensioning devices with robotic systems and exploring the potential role of artificial intelligence (AI) in interpreting intraoperative data. By synthesizing historical innovations with contemporary evidence, we aim to clarify the clinical utility of different tensioning strategies, highlight approaches to improve soft-tissue balancing, and ultimately optimize patient outcomes in TKA.

## Methods and data sources

We conducted a comprehensive literature search of the PubMed, Embase, and Web of Science databases from inception up to March 2025 for English-language studies on soft-tissue balancing in TKA and related tensioning devices. This article is explicitly a narrative review rather than a systematic review or meta-analysis and therefore does not follow the Preferred Reporting Items for Systematic Reviews and Meta-Analyses (PRISMA) or International Prospective Register of Systematic Reviews (PROSPERO) protocols. Both classic foundational articles and recent high-quality studies were included to ensure a thorough historical overview and comprehensive evaluation of the current evidence. Articles were selected if they were particularly relevant or historically significant or contributed meaningfully to the clinical context of the topic. Emphasis was given to information gained from relevant studies and practical information of interest to the general orthopedic readership.

We first present a historical narrative of soft-tissue tension gauging methods in three developmental stages (experiential, mechanical, and digital). Within the digital stage, we differentiate passive versus active tensioning devices. We then provide a comparative discussion of the approaches, supported by clinical evidence. Finally, we propose a clinically useful algorithm and discuss future developments, including AI integration.

## Stage 1 (1970s): Experiential tools

**Fig. 1:**
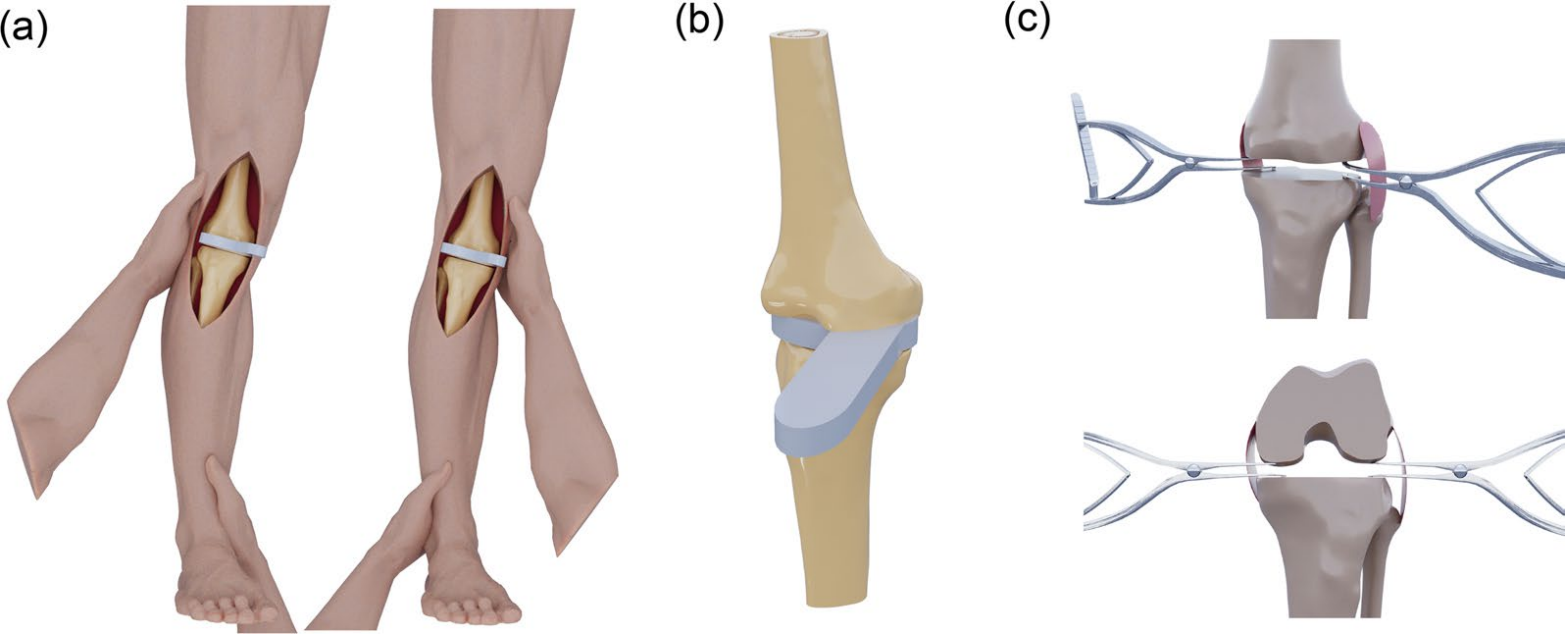
Experiential stage of soft-tissue tension assessment: **A** valgus and varus stress tests for medial–lateral knee stability evaluation; **B** spacer block technique for assessing extension and flexion gaps; and **C** lamina spreader technique used to measure and maintain joint spacing

As early as the 1970s, gauging soft-tissue tension became a surgical challenge, and surgeons relied primarily on empirical techniques and personal judgment to achieve ligament balance (Fig. 1A). It has been a thorny issue to decide when to discontinue from the soft-tissue release to correct the valgus deformity while avoiding knee instability in the opposite direction. Then, in 1974, Michael Freeman and William Day at Imperial College developed the first dedicated tensor instrument for knee balancing, which could be inserted into the two compartments and independently separate the tibial and femoral condyles while checking the overall limb alignment (“hip‒knee‒ankle” alignment) [1]. With this tensor, surgeons could gradually release soft tissue until the correct alignment was obtained [1].

Since then, various tools and tensor instrumentation designs have emerged to facilitate appropriate soft-tissue release and achieve soft-tissue balance (Table 1), including spacer blocks and lamina spreaders (Fig. 1B, C). Spacer blocks of known thickness were frequently utilized during primary TKA to evaluate gap balance, correct deformities, and ensure soft-tissue balance [9]. Similarly, lamina spreaders could be applied medially and laterally to distract the knee joint, assess the symmetry of the extension gap, and evaluate soft-tissue balance. However, these devices and experiential methods provided only a qualitative sense of balance and thus had significant limitations. The tools were bulky and unreliable and were unable to get rid of operator subjectivity. Furthermore, these methods for assessing soft-tissue tension and balancing relied heavily on the “best judgment” of the surgeon to determine what “feels” or “seems” like a balanced knee. Minor differences in how much force was applied during a stress test or how a spacer block was leveraged could change the perceived gap tightness.

**Table 1:** Summary of tensioning devices for soft-tissue tension gauging in total knee arthroplasty

Developmental history	Soft-tissue tension gauging tools	Designers	Manufacturer	Physiological or not	Offset-type or not	Analytical-type or not
Stage 1 (1970s): experiential tools	Tensor	Michael Freeman	—	No	—	No
	Spacer blocks	—	—	No	—	No
	Lamina spreaders	—	—	No	—	No
Stage 2 (1990s): mechanical tensors	Xcelerate knee balancer	Balancer Study Group	Stryker	No	No	No
	V-STAT	—	Zimmer	No	No	No
	Balansys	—	Mathys AG	No	No	No
	Offset Repo-tensor	—	Zimmer	Yes	Yes	No
	FuZion tensor	—	Zimmer	Yes	Yes	No
	BalAnswer	—	DePuy	No	No	No
	TIPI	—	Amplitude	No	No	No
	Balanceur	—	Amplitude	No	No	No
	CORES device	—	Stryker	—	—	No
Stage 3 (2000s): Passive digital tensors	e-Knee system	Darryl D. D’Lima	—	Yes	No	No
	eLIBRA	—	Zimmer	Yes	No	Yes
	VERASENSE	OrthoSensor	Stryker	Yes	No	Yes
	Gap Balancer	—	Yiemed	Yes	No	Yes
Stage 3 (2000s): Active digital tensors	Digital tensor	—	DynAccurate	Yes	Yes	No
	Newton	—	Exactech	Yes	No	Yes
	BalanceBot Ligament Tensioner	—	Corin	No	No	Yes
	CORI Digital Tensioner	—	Smith & Nephew	No	No	Yes
	Balance Solver	—	Tinavi	Yes	Yes	Yes

At this stage, although surgeons typically assessed soft-tissue balance qualitatively through manual intraoperative trialing (e.g., varus and valgus stress tests) or experimental tools, such as alignment rods, spacer blocks, lamina spreaders, or traditional tensors [2], true quantification of soft-tissue tension remained unavailable. Without objective measures, achieving reproducible soft-tissue balance remained an art as much as a science.

## Stage 2 (1990s): Mechanical tensors

By the 1990s, attention had turned to designing instruments that could provide quantitative measurements of soft-tissue balance. The goal was to eliminate much of the guesswork associated with manual tensioning by using mechanical devices that applied a known force and measured the resulting joint gap or angle. The Xcelerate knee balancer (Stryker Howmedica Osteonics, Allendale, NJ), developed by the Balancer Study Group, was an early example of a dedicated mechanical tensioner for TKA. This device could distract the knee joint via a ratcheting system and measure soft-tissue imbalance by assessing the angulation of a pivoting plate that contacts the femoral condyles [10, 11]. Asymmetry in soft-tissue tension caused the pivoting plate to tilt, and the measured degree of tilt could thereby be used to quantify the mediolateral imbalance within the gap. Consequently, unlike spacer blocks, which rely on trial components and joint compression, this tensioning device could independently quantify soft-tissue imbalance without relying on compressive passive loads through the knee [12]. This allowed the measurement of soft-tissue tension in a controlled and consistent manner, independent of variations in the force applied by the surgeon or interaction of the joint surfaces.

**Fig. 2:**
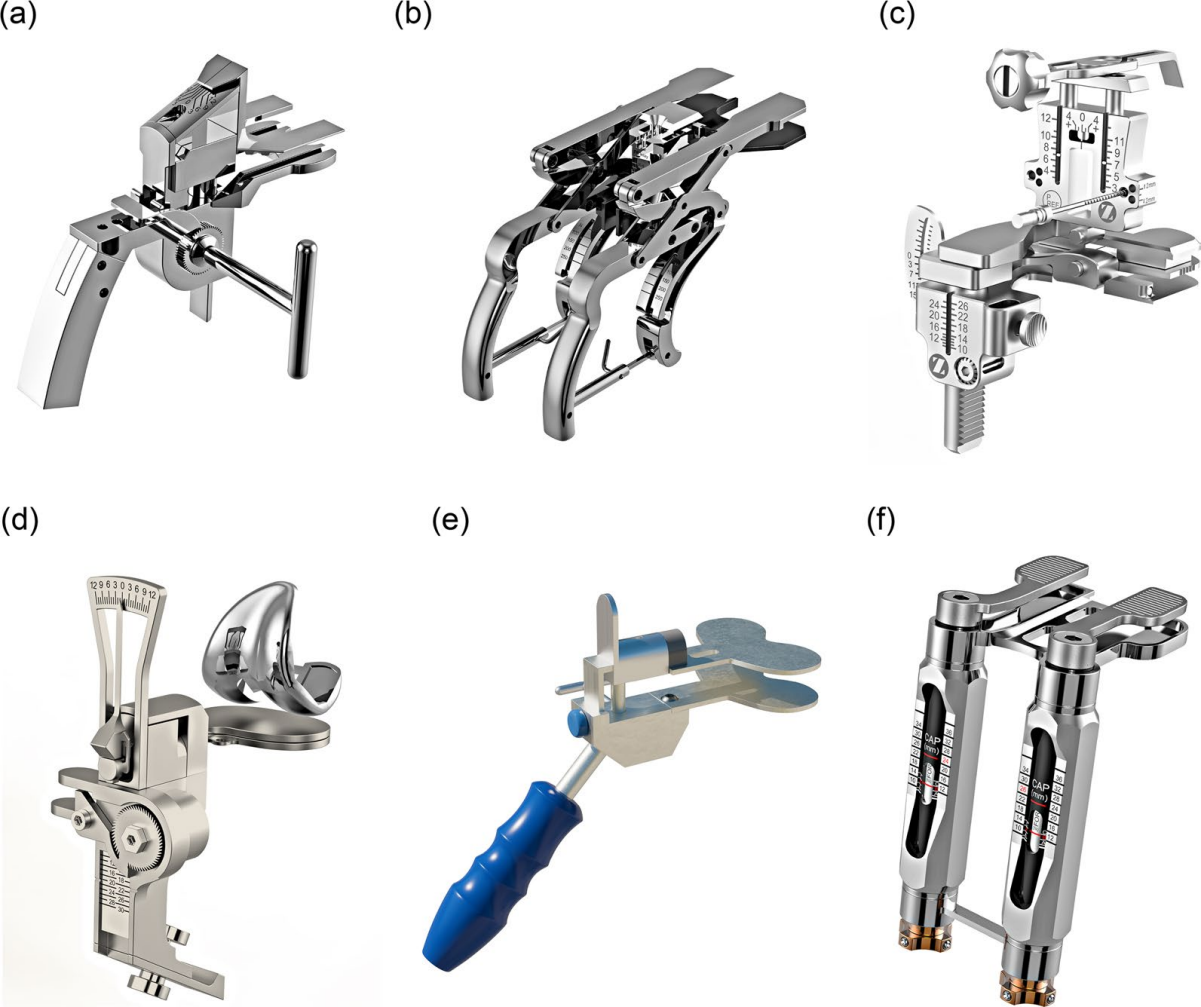
Mechanical tensor stage: **A** Xcelerate Knee Balancer (Stryker); **B** Balansys tensor (Mathys); **C** FuZion tensor (Zimmer); **D** True Tensor (Biomet Japan); **E** Balanceur (Amplitude); **F** BalAnswer tensor (DePuy)

Subsequently, several similar parametric mechanical soft-tissue tensioning devices were developed, including the V-STAT variable soft-tissue alignment tensor (Zimmer, Warsaw, IN, USA) [13, 14], Balansys (Mathys AG, Bettlach, Switzerland) [15], Offset Repo-tensor (Zimmer) [16], FuZion tensor (Zimmer) [17], BalAnswer (DePuy, Warsaw, IN, USA) [18], TIPI (Amplitude, Valence, France) [19], Balanceur (Amplitude) [19], CORES device (Stryker) [20], Tensor (Medacta International, Switzerland) [21], and others (Fig. 2). These mechanical tensors share common principles and possess the following key features [22]:

1. A vertical distractor that can distract the knee joint and have a measured load;
2. A teeter-totter plate or seesaw component that can rock mediolaterally to reveal imbalance;
3. A gauge (mechanical scale, dial, or caliper) that can be used to measure the gap distance or angular deviation between the femur and the tibia.

Despite differences in specific designs, these tensors were designed to separate the femur and tibia by applying controlled distraction forces equally to both the medial and lateral compartments, either independently or simultaneously. They all sought to quantify the tensioning force parametrically, as well as the femorotibial angle or mediolateral angular displacement. Mechanical tensors brought several advantages over purely experiential techniques. They enabled surgeons to apply consistent forces to the joint, thereby standardizing the tension applied to the knee during evaluation. Additionally, they provided visual or numeric feedback, as surgeons could read how many millimeters of asymmetry were present rather than relying purely on feel.

These improvements notwithstanding, limitations persisted. Mechanical tensors generally measured the knee under static, non-weight-bearing conditions similar to previous methods—the leg was often in the air or on the table at a specific flexion angle. They indicated imbalance at that moment but did not account for dynamic factors such as muscle forces or joint motion. Many mechanical devices measured balance only at a few key angles (typically extension and 90° of flexion). They also added steps and instrumentation to the workflow, and some designs were cumbersome or time-consuming to use. As a result, adoption in routine practice was not universal; some surgeons continued to rely on simple tools or intuition, especially when mechanical tensors were not readily available. Clinical evidence related to the mechanical tensors was rather limited, and the improvements were always marginal when compared with existing techniques such as spacer blocks or laminar spreaders [23]. Nevertheless, the mechanical stage established the principle that soft-tissue tension could be measured and quantified intraoperatively [24], laying groundwork for digital innovations.

## Stage 3 (2000s): Digital and analytical tensors

As the twenty-first century has advanced, rapid progress in sensors, electronics, microelectromechanical systems, and computer processing has paved the way for more sophisticated, automated, and precise tensiometers. Further developments in tensors are trending toward electronic, wireless radiofrequency transmission, and digital displays. The Balancer Study Group has predictably outlined the development of tensors, as they experimentally tested the electronic measurement of separation gaps and the femoral‒tibial angle [11]. Contemporary pioneers have dedicated themselves to measuring in vivo knee forces using pressure sensors, ultimately leading to the creation of next-generation tensors—digital tensors. The digital stage of TKA tensioners is characterized by devices that incorporate pressure sensors and/or electromechanical elements to quantify the femorotibial contact pressure and/or measure separation gaps simultaneously. These digital systems aim to provide real-time analytics and guidance, transforming balancing from a qualitative exercise into a data-driven process. We further divide the digital stage into two subcategories: passive digital tensors and active digital tensors, reflecting fundamental differences in their operation and capabilities.

### Passive digital tensors

**Fig. 3:**
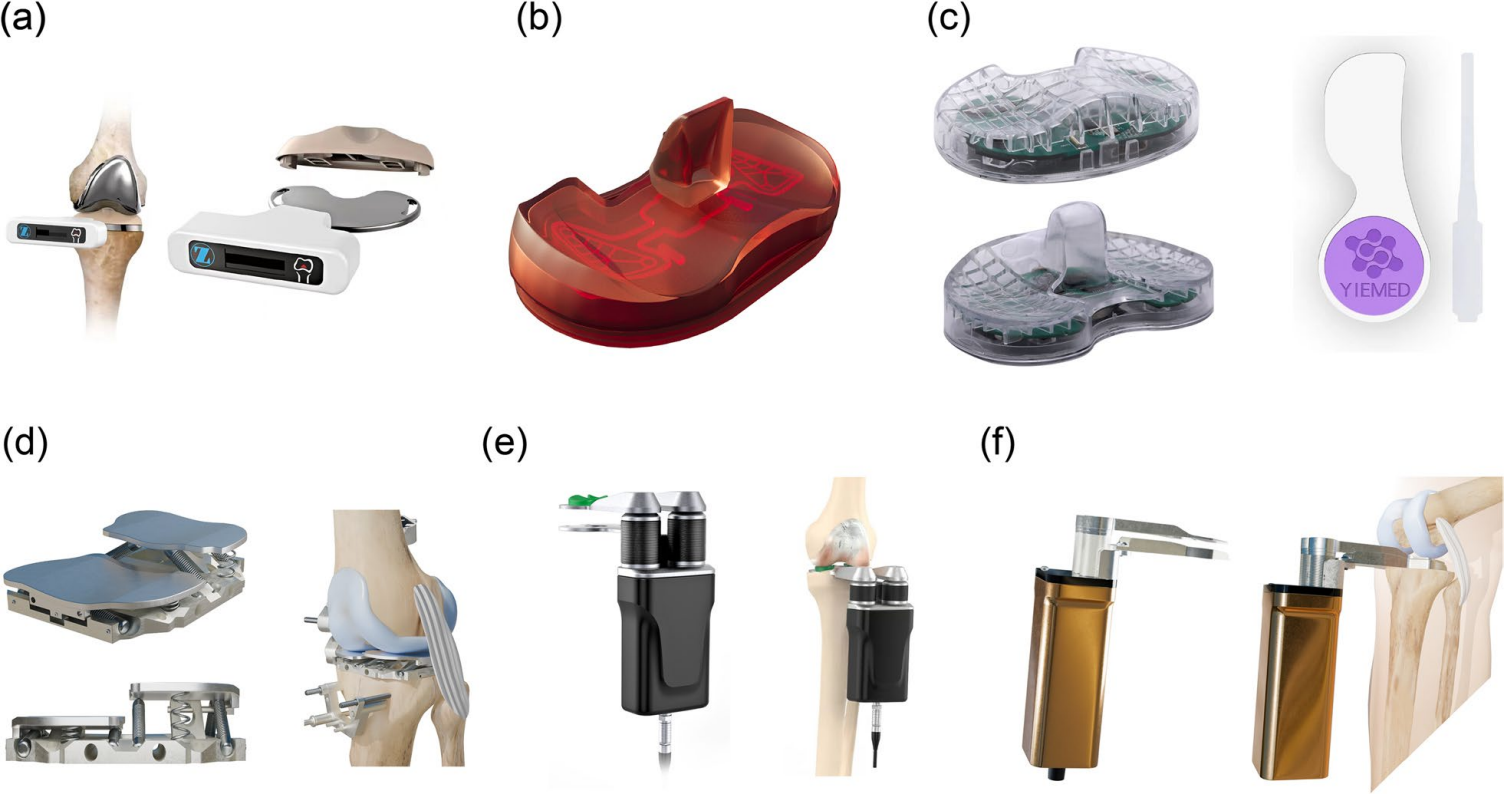
Digital tensor stage: **A** eLIBRA Dynamic Knee Balancing System (DKBS) (Zimmer); **B** VERASENSE Knee System sensor (OrthoSensor); **C** Gap Balancer system (Yiemed); **D** Newton Balanced Knee System (Exactech); **E** BalanceBot Robotic Ligament Tensioner (Corin); **F** Balance Solver digital tensioning device (Tinavi)

Passive digital tensors typically refer to sensor-equipped devices that do not actively apply force to the joint but instead passively measure the forces generated during standard surgical trialing. The most common form is an instrumented tibial trial insert that is used in place of the normal plastic trial during TKA balancing. These devices contain load sensors that detect the force in the medial and lateral compartments when the surgeon brings the knee through motion or applies stress. Various types of passive digital tensors have been employed in clinical practice, including but not limited to the e-Knee system [25], the eLIBRA Dynamic Knee Balancing System (DKBS) (Zimmer) [26], the VERASENSE Knee System sensor model (OrthoSensor, Dania Beach, FL, USA) [27], and the Gap Balancer (Yiemed, Shandong, China) [28] (Fig. 3).

#### e-Knee

The e-Knee is an advanced total knee prosthesis, including transducers (load cells), a transmitter, and an antenna, that can be inserted into the tibial canal to measure tibial forces [29]. Powered by an inductive coil, the e-Knee collects and transmits data to a computer, where it is converted into a readable format [28]. This device enables direct in vivo measurement of tibiofemoral compressive and tensile forces throughout the ROM, thereby facilitating improvements in implant design, enhancing intraoperative decision-making, optimizing postoperative rehabilitation protocols, and informing the development of assistive devices [30].

#### eLIBRA

The eLIBRA DKBS was initially developed by Synvasive Technology and subsequently modified by Zimmer-Biomet following their acquisition [31]. It comprises a resterilizable instrument tray (specific to the implant) and a disposable sterile electronic force sensor. The reusable stainless steel eLIBRA femoral component features a low-profile, implant-like articular shape that allows for patellar reduction during measurement. The single-use eLIBRA force sensor integrates a thick-film sensor element, microprocessor, accelerometer, battery, and organic light-emitting diode (OLED) display [32, 33]. The eLIBRA force sensor is factory-calibrated and capable of displaying force on a scale from 0 to 20, with each unit representing approximately 15 N [32, 33]. Consequently, the eLIBRA provides objective, real-time measurement of the forces across the medial and lateral compartments while allowing dynamic, controlled adjustment of femoral component rotation [34]. Moreover, this device can also be utilized during trialing to evaluate soft-tissue tension and assist surgeons in selecting an appropriate polyethylene insert thickness [34]. D’Angelo et al. conducted a short-term follow-up and reported on the simplicity of the instrument and the reproducibility of soft-tissue balancing [35].

#### VERASENSE

The VERASENSE Knee System (developed by OrthoSensor, acquired by Stryker in 2021) was cleared by the Food and Drug Administration (FDA) in 2009 and is currently integrated into the Mako Smart Robotic Workflow, providing a comprehensive, data-driven intraoperative feedback mechanism [36]. VERASENSE is a single-use, wireless device that transmits evidence-based data to an intraoperative monitor, facilitating informed decision-making regarding ligamentous soft-tissue balance and implant position in real time [37]. The sensor relies on a “smart” tibial trial, which is composed of pressure nanosensors, microprocessors, and miniaturized integrated circuits connected wirelessly to a portable graphic display unit that presents real-time data for the interpretation by the surgeon [38]. When inserted as a tibial trial component and used with patellar relocation, this sensor accurately measures peak loads in the medial and lateral tibiofemoral compartments throughout the ROM and locates the contact points between the femoral and tibial components, assisting in tibial tray rotation adjustments and optimizing tibiofemoral articulation [39].

The first-generation VERASENSE smart trial did not provide any alignment information, requiring surgeons to rely on conventional instrumentation or computer-assisted navigation to obtain accurate component alignment. Subsequently, the upgraded version incorporated accelerometers to ascertain the tibial varus‒valgus angle and lower limb mechanical axis alignment. Consequently, the tibial axial alignment and overall lower limb mechanical alignment are available for consideration when deciding on bone recutting or targeted soft-tissue release [40]. OrthoSensor has partnered with leading joint replacement manufacturers, including Smith & Nephew, Stryker, and Zimmer Biomet, allowing the smart trials to be compatible with various implant systems by adopting standard trial device configurations [41]. Despite this smart trial providing satisfying soft-tissue balance and improved patient outcomes [38, 39, 42], clinical trials and systematic reviews have demonstrated no difference in ROM, reoperation rate, or functional outcomes compared with manually balanced TKA [42–45].

#### Gap Balancer

Similar to the VERASENSE sensor, the Gap Balancer (Yiemed, Shandong, China) is a wireless electronic tibial trial developed to quantify medial and lateral compartment pressures during TKA [28]. The sensor accommodates gaps greater than 9 mm, and a 1 mm or 2 mm thickener can be used to adapt to different gaps for accurate measurement [46]. Additionally, Yiemed developed an electronic gasket for measuring tibiofemoral force in unicompartmental knee arthroplasty (UKA). The pressure sensor is fabricated in the shape of a mobile-bearing spacer for the Oxford UKA prosthesis, featuring a data cable that connects to a computer for data acquisition. The gasket can be inserted into the medial compartment, allowing contact pressure to be measured at five predetermined flexion angles (0°, 20°, 45°, 90°, and 110°). The thickness of the sensor is 6 mm, and 1 mm metal shims can be used on the tibial side to ensure an appropriate fit and reliable measurements [47].

#### Advantages and disadvantages

Passive digital tensors offer several significant advantages. They provide direct, real-time in vivo measurements of tibiofemoral forces across both the medial and lateral compartments throughout the ROM while accounting for patellofemoral kinematics, which offers surgeons precise intraoperative feedback to guide soft-tissue release and facilitate intercompartmental load balance. Another practical advantage is their cost-effectiveness, with disposable sensors priced at approximately $500 per case [48].

However, passive digital tensors have notable disadvantages. Passive digital tensors did not improve clinical outcomes or patient-reported outcome measures (PROM) as expected [44, 49], which may be attributed primarily to their static measurement methodology, as the sensors provide force data only under specific nonphysiological loading conditions. In other words, the use of sensor-guided tibial trials yields balanced force measurements solely in a “static” context, lacking the dynamic loads generated by muscle contractions and weight-bearing conditions encountered in daily activities [50]. Therefore, the intraoperative measurement obtained from passive digital tensors represents merely a static “snapshot” of soft-tissue balance, which is insufficient to predict dynamic, real-life soft-tissue tension during daily activities [51, 52].

Therefore, a balanced medial–lateral compartment reading from passive digital tensors does not necessarily indicate genuine soft-tissue balance. It neither guarantees balanced forces on the soft tissue nor ensures uniform intra-articular pressure across the prosthetic surfaces under functional conditions, as ligament forces measured passively differ significantly from dynamic forces during actual knee movements [51, 52]. Consequently, these limitations prompted the development of active digital tensors, which are capable of applying independent or asynchronous distraction forces to the medial and lateral compartments dynamically, thereby providing a more accurate representation of soft-tissue balance throughout the ROM.

### Active digital tensors

Active digital tensors can apply controlled distraction forces to both the medial and lateral compartments, effectively simulating ligamentous or muscular contraction and weight-bearing conditions during the intraoperative assessment of soft-tissue balance. Representative active digital tensor systems currently include the Digital tensor system (DynAccurate, Stryker, Japan) [53], the Newton Balanced Knee System (Exactech, Gainesville, FL, USA) [54], the BalanceBot Robotic Ligament Tensioner (Corin, Tampa, FL, USA) [55], the CORI Digital Tensioner (Smith & Nephew Healthcare, Watford, England, UK) [56], and the Balance Solver (Tinavi, Beijing, China) [57] (Fig. 3).

The Digital tensor is a microelectronically modified offset seesaw-type mechanical tensor comprising load cells, angle sensors, and gap sensors. This device features dedicated femoral and tibial side trays, with a center peg underneath the tibial tray designed to engage the tibial peg hole, allowing for rotational alignment adjustments of the tibia [58]. This tensor can apply continuous joint distraction forces ranging from 10 to 60 lb while simultaneously measuring and automatically recording three critical parameters: the applied force, femorotibial angle, and joint gap size [58]. Gap assessments can be performed in both extension and flexion with the patellofemoral joint reduced by temporarily placing two stitches proximal and distal to the patella [53]. Although the utilization of this active tensor has demonstrated improved soft-tissue balance and more consistent knee alignment outcomes in TKA, these improvements have not consistently achieved the minimum clinically important difference in postoperative clinical outcomes [53].

#### Newton balanced knee system

The Newton Balanced Knee System (Exactech) is designed to provide real-time dynamic soft-tissue analytics, preresection operative insights, and full-range personalized planning to simplify and enhance reproducibility in balanced TKA procedures [59]. This system features an intra-articular distractor, which can be inserted into the joint space between the proximal tibia resection and the native femur after the first tibial resection, maintaining consistent joint tension without patellar eversion [60]. The distractor features two independent mechanically actuated compartments designed to apply a quasiconstant distraction force (nominally set up at 20 lb per compartment) regardless of the joint gap variations, which is achieved by balancing two different internal mechanisms: a Hookian spring that increases the force output as it is compressed and a non-Hookian spring mechanism that is defeated as it is compressed.

The Newton Knee integration, which is exclusively integrated into the Exactech GPS platform, provides surgeons with real-time dynamic soft-tissue analytics, valuable preresection operative insights, and personalized surgical planning across the ROM [60]. This integrated approach leverages Global Positioning System (GPS) technology to provide optimal balance in a reproducible manner [54]. Although preliminary studies have indicated statistically and clinically significant improvements in functional scores compared with those of the control, further validation from independent, nondesign surgeons remains necessary to confirm these promising findings and establish broader clinical applicability [61].

#### BalanceBot

The BalanceBot Robotic Ligament Tensioner (also called the OMNIBotics Active Spacer) was initially developed by OMNI as an innovative robotic tensioning and sensor-based device to augment soft-tissue balancing during TKA and was released in 2017 [62]. In 2019, the Corin Group (Cirencester, UK) acquired the OMNI Orthopaedics [63]. Designed specifically to integrate with the OMNIBot robotic platform, this system functions similarly to a robotic laminar spreader, precisely applying controlled distraction forces to the medial and lateral compartments to measure joint gaps dynamically throughout the ROM [55].

The BalanceBot system utilizes two independent motorized actuators integrated with force sensors. A tibial baseplate that matches the size and profile of the selected tibial tray size, along with two upper arms, is attached to the BalanceBot device, enabling simultaneous joint distraction force application and accurate gap measurement [55]. Controlled by the OMNIBotics system, it operates in either force mode or position mode to measure the gap distance under controlled ligament tension or to measure the medial and lateral compartment loads for a controlled gap distance, respectively. After tibial resection, the device applies patient-specific, symmetrical distraction forces (typically 70–100 N), customized according to individual patient weight and soft-tissue characteristics [55].

The captured soft-tissue tension and gap distance can be utilized intraoperatively for implant placement to ensure proper alignment and soft-tissue balance, often eliminating the need for additional soft-tissue release. After femoral resection and trial component placement, the robotic tensioner is reinserted into the joint, and the postoperative soft-tissue tension and gap profiles are collected throughout the ROM with the patella reduced, using the same loading profile as the prefemoral resection gap acquisition. This enables surgeons to measure soft-tissue balance intraoperatively; additionally, a predictive balance function has been reported to assist ligament tension before bone resection, thereby minimizing the extent of soft-tissue release needed to achieve a balanced knee [64]. Less soft-tissue releases during TKA may decrease local inflammation, postoperative pain, and recovery time to functional milestones [64]. Preliminary clinical studies have demonstrated improved intraoperative soft-tissue balancing using this robotic tensioning device; however, current evidence remains inconclusive, and further robust studies are needed to confirm meaningful improvements in clinical and patient-reported outcomes [65–67].

#### CORI Digital Tensioner

The CORI Digital Tensioner (Smith & Nephew Inc., Memphis, TN, USA) is a recently released handheld device designed to evaluate ligament laxity during robot-assisted knee arthroplasty, even prior to bone resection [68]. This tensioning device applies a surgeon-defined, quantifiable force to distract the medial or lateral compartment, consistently maintaining ligament tension while providing objective gap data for surgical planning and execution. This technique enables surgeons to precisely quantify native joint laxity and achieve optimal ligament tension, significantly reducing variability in knee balancing during surgery. This device is designed for robotic tensioning to enhance robot-assisted surgical procedures and provides a software interface to help surgeons select their preferred target force value (50 ± 10 N, 100 ± 10 N, or 150 ± 10 N) [69]. Since its release in 2023, preliminary clinical evidence from a small clinical case series has shown that the CORI Digital Tensioner significantly reduces the variability of tensioning by 64% when compared with the manual technique [70].

#### Balance Solver

The Balance Solver is an innovative digital tensioner featuring a tibial base plate and dual femoral paddles for knee joint insertion. Each upper paddle connects independently to an electromotor via a dedicated paddle connector, allowing the controlled application of separate distraction forces to the medial and lateral compartments. Additionally, integrated sensors positioned below the electromotor assembly measure soft-tissue tension forces precisely [57]. Joint gap measurements are determined by built-in displacement controllers, which assess the distance from each femoral paddle to the tibial baseplate. Powered by the electromotor, the device can independently apply adjustable distraction forces ranging from 30 to 120 N (increasing by intervals of 5 N), while measuring joint gaps of 4–25 mm, and tension forces of 0–120 N. Design verification has demonstrated excellent measurement accuracy, with gap size precision within 0.6 mm and soft-tissue tension force accuracy within 1.5 N [57].

Furthermore, the Balance Solver interfaces seamlessly with the Gap–Alignment–Force (GAF) system, enabling surgeons to select preferred target force intervals (e.g., 30–90 N) or preferred measurement models (e.g., the Solver model). The digital tensioner applies independent, asynchronous distraction forces to the medial and lateral compartments. It automatically captures real-time separation gap sizes and corresponding soft-tissue tension forces. These comprehensive data points, comprising distraction forces, gap sizes, and soft-tissue tensions, are wirelessly transmitted via Bluetooth to the GAF system and integrated into intuitive graphical representations. This facilitates immediate intraoperative decision-making, guiding surgeons precisely in performing surgical adjustments such as targeted bone recutting or specific soft-tissue release [57].

#### Advantages and disadvantages

Active tensioners directly address some shortcomings of passive sensors and offer substantial advantages by applying consistent, controlled distraction forces independently to both the medial and lateral compartments of the knee. These devices accurately measure joint gaps, transmit the data wirelessly and provide surgeons with real-time, visually intuitive feedback to guide intraoperative adjustments.

However, a key disadvantage is that most active digital tensors apply constant distraction forces, despite the soft-tissue envelope around the knee being viscoelastic and deformable rather than rigid and inextensible. Although the BalanceBot addresses this by applying lower forces in flexion than in extension to better replicate physiological ligament tension patterns, it still applies symmetrical forces to the medial and lateral compartments, potentially oversimplifying the natural asymmetry of soft-tissue behavior. In contrast, the Balance Solver can dynamically apply independent distraction forces to the medial and lateral compartments, accurately characterizing the medial and lateral viscoelastic properties and generating detailed load‒deformation curves. This approach provides surgeons with more precise and comprehensive quantitative data for intraoperative decision-making and personalized soft-tissue balancing [71].

## Comparison of tensors

To better appreciate the practical implications of each tensioning approach, it is useful to conduct a side-by-side comparison of their performance, advantages, and limitations. The baseline characteristics and key features of representative devices are summarized in Table 1. On the basis of their characteristics and design philosophy, mechanical tensors can be further categorized as follows [22]:

- *Physiological or nonphysiological:* The former can work in both flexion and extension, maintaining the patella in its physiological position.
- *Offset or non-offset:* The former consists of two plates connected to the extra-articular main body via an offset connection arm through a medial parapatellar arthrotomy, allowing for the reduction of the patellofemoral joint while performing measurements.
- *Pure mechanical, elastic, or hydraulic:* The tensor applies and measured the forces through a screw gear, spring, or liquid column, respectively.
- *Analytical or non-analytical:* The former can provide real-time dynamic measurements, integrate with computer systems or robotic platforms, and perform advanced data analysis to guide intraoperative decisions, whereas the latter offers only basic, static measurements without further data interpretation or analytical support.

Experiential techniques rely heavily on the skill of the surgeon and tactile feedback from tools, such as spacer blocks and lamina spreaders, or simply stressing the knee by hand. The strength of experiential balancing is its simplicity and ubiquity, but outcomes remain subjective and inconsistent. Mechanical tensors introduce objectivity and repeatability by quantifying joint gaps and applied forces, thus improving intraoperative gap symmetry and accuracy in soft-tissue balancing. However, mechanical tensors measure soft-tissue tension under static, controlled conditions at fixed knee angles, limiting their ability to represent dynamic knee function, and clinical evidence supporting improved patient outcomes remains sparse, although these devices have facilitated advancements toward digital tensors. Passive digital tensors provide precise real-time intra-articular force measurements, allowing surgeons to finely adjust soft-tissue balance across multiple angles, potentially reducing extreme postoperative imbalances. However, passive digital tensors capture only static loading conditions and fail to reflect dynamic knee behavior. In contrast, active digital tensors integrate quantification with dynamic, physiologic simulations, offering detailed, continuous intraoperative data. They enable surgeons to address complex balance scenarios across the full ROM and facilitate soft-tissue balancing surgical approaches.

**Fig. 4:**
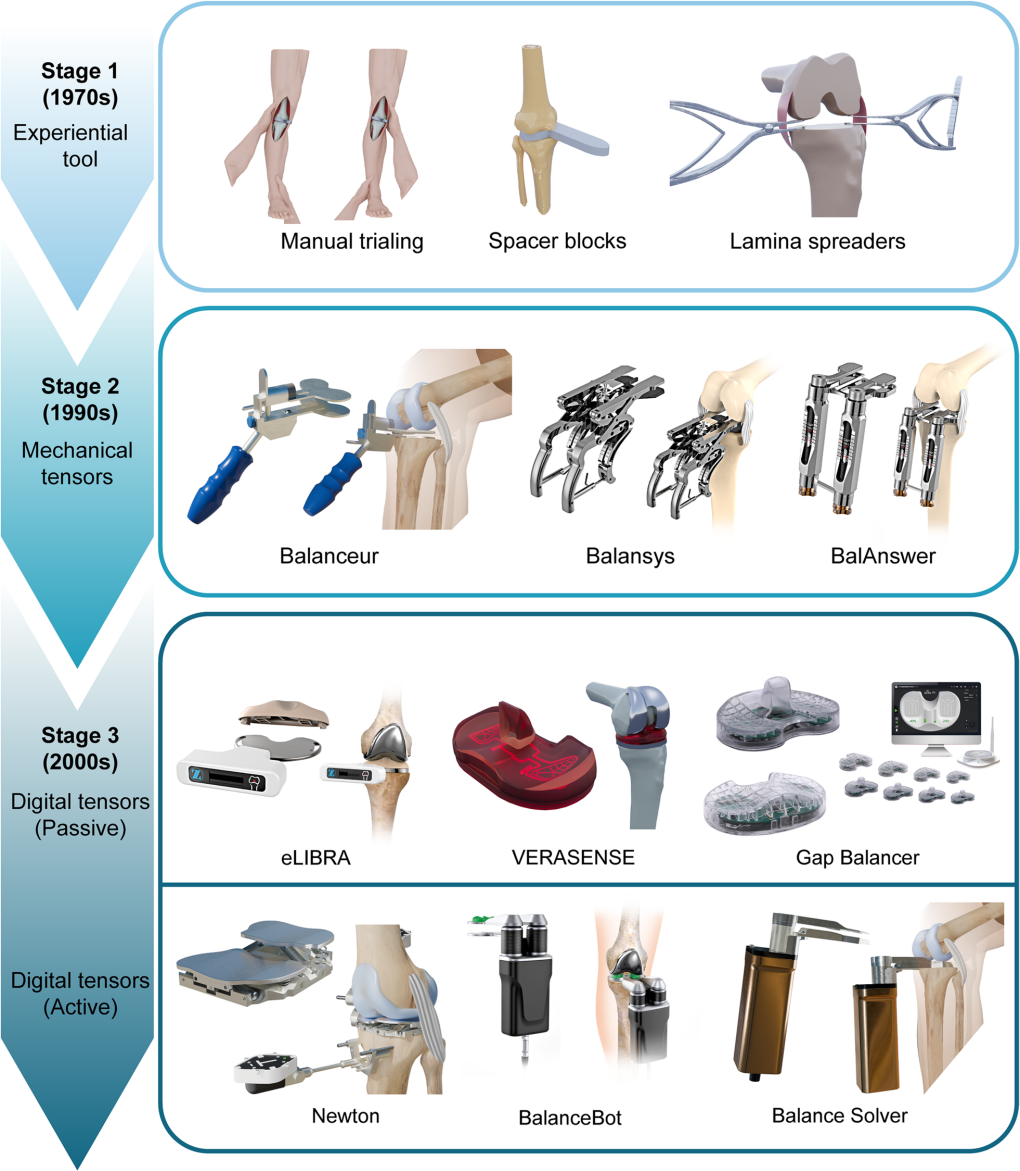
Historical overview illustrating the evolution of soft-tissue tension assessment methods in total knee arthroplasty, highlighting the transition from manual techniques to advanced digital systems

Over 50 years, soft-tissue tension measurement in TKA has evolved from the experiential stage to the mechanical tensor stage and then to the current digital and analytical tensor stage (Fig. 4). This progression has transformed our historical reliance on tactile feedback into objective and consistent measurements of tension forces and joint gaps, resulting in enhanced repeatability and consistency of tension force measurements compared with manual techniques. In comparison, each tensor design has a clinical niche and associated trade-offs regarding precision, complexity, and usability. Analytical, physiological, offset-type active digital tensors are positioned optimally and combine accurate physiological simulation, precise force modulation, and advanced data analytics, significantly enhancing clinical outcomes in soft-tissue balancing.

## Preliminary clinical evidence

Clinical evidence regarding the effectiveness of various soft-tissue tensioning methods in TKA varies significantly across device types. Despite their simplicity and widespread adoption, experiential techniques exhibit substantial variability due to their subjective nature. Studies consistently indicate that manual assessment frequently misses moderate soft-tissue imbalances, potentially contributing to inconsistent postoperative outcomes [72]. Mechanical tensioners have introduced objectivity and reproducibility into TKA, enhancing intraoperative gap symmetry and soft-tissue balancing accuracy. However, these tensioners are not widely used today, and clinical evidence directly linking mechanical tensioners to improved patient outcomes remains limited [23, 73, 74].

The clinical evidence for passive digital tensors demonstrates reliable quantitative intraoperative data collection, resulting in objectively balanced knees. Nevertheless, multiple randomized controlled trials and systematic reviews confirmed that although soft-tissue balance objectively improved with these devices, there was no difference in patient-reported outcome measures (PROM), such as the Knee Society Score (KSS) and the Western Ontario and McMaster Universities Osteoarthritis Index (WOMAC), or patient satisfaction at clinical follow-up [42–44, 49, 75, 76]. The lack of outcome improvement may be attributed primarily to the static nature of intraoperative soft-tissue balancing, which limits the translation of intraoperative soft-tissue balance into meaningful functional improvements. Active digital tensors, which integrate real-time dynamic measurements with physiologic load simulations, have shown the most promising preliminary clinical evidence. Among these platforms, the BalanceBot platform is the most extensively studied. Initial clinical studies and systematic reviews suggest improvements in surgical planning and modest improvements in accuracy and superior PROM scores compared with conventional and navigation-assisted techniques [62, 66, 67, 76, 77]. While these findings are encouraging, high-quality long-term clinical data remain limited. Further robust, larger-scale studies with extended follow-ups are essential to conclusively demonstrate the sustained clinical benefits and meaningful improvement in patient outcomes associated with active digital tensor systems.

## Future perspective

Current tensioning devices remain imperfect, and the conflicting evidence indicates a pressing need for continuous innovation and improvement [44, 76, 78, 79]. The next generation of tensioning devices may integrate emerging technologies such as shear wave elastography [80] and broaden their application beyond TKA to measure soft-tissue envelopes in procedures on other joints, such as total hip arthroplasty. Simultaneously, many current and emerging tensioners are designed as robotic instruments that integrate with robotic systems to enhance multisource information perception. The BalanceBot, CORI Digital Tensioner, and Balance Solver are representative examples of devices that effectively embody this integrated tensioner and robotic approach.

**Fig. 5:**
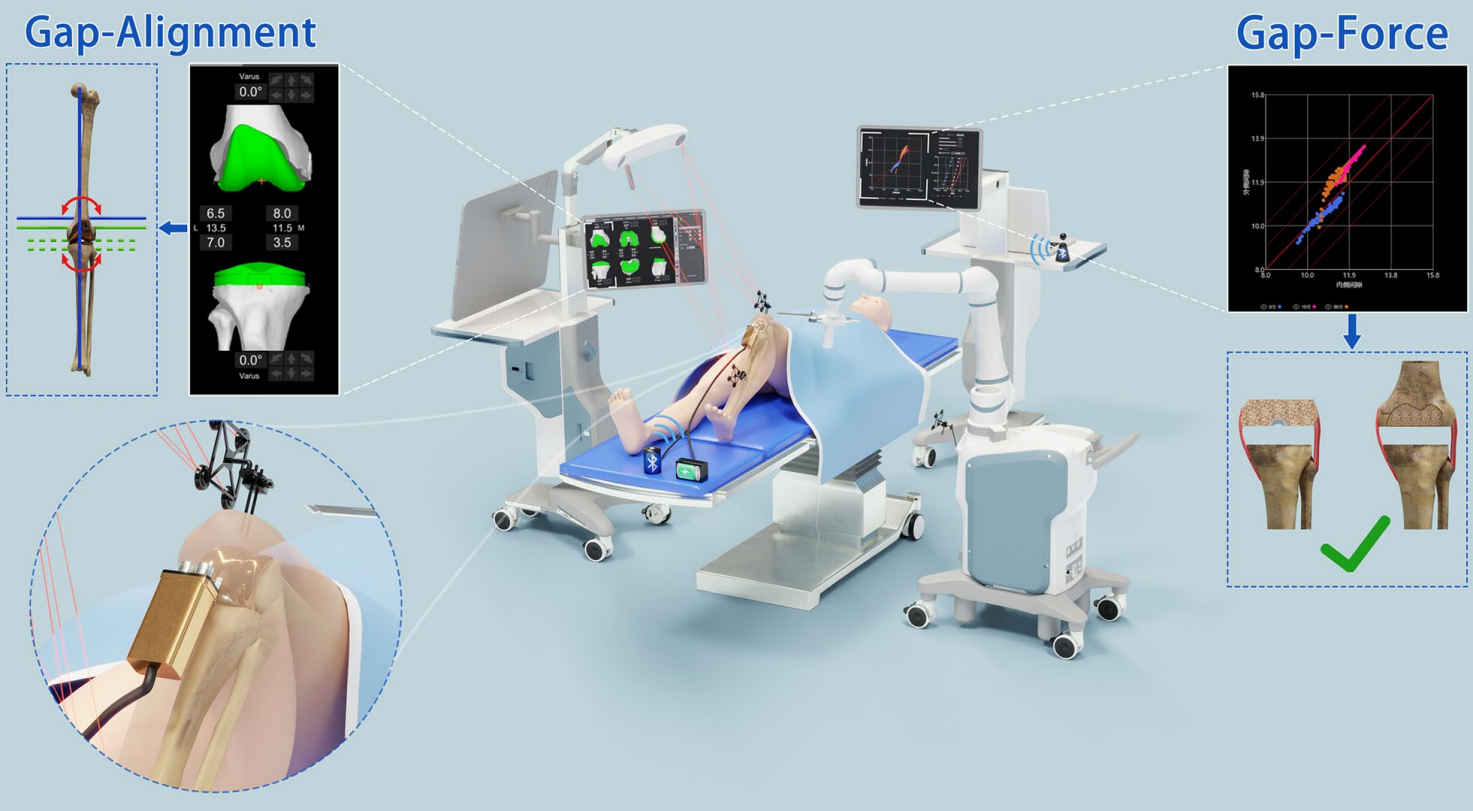
Conceptual illustration showing the integration of digital tensioning technology with robotic surgical systems to comprehensively address the Gap–Alignment–Force balance triad, critical for optimal outcomes in total knee arthroplasty

Achieving a balanced knee in TKA requires a delicate balance of three key elements: gap balance, ideal lower limb alignment, and optimized soft-tissue balance. Therefore, integrating digital tensiometers with robotic systems represents the most promising direction for advancement, as this combination may effectively address all the components of the Gap–Alignment–Force triad (GAF triad), which is essential for optimal knee balance. The integration of digital tensiometers with robotic systems appears highly promising, as digital tensiometers help achieve appropriate tension of the soft-tissue envelope, whereas robotic systems help optimize lower limb alignment and implant alignment, ensuring balanced flexion‒extension gaps without mediolateral tightness or laxity (Fig. 5). However, seamless integration faces technical hurdles, including effective data communication, real-time intraoperative computations, and dynamic multivariate optimization. As these systems generate extensive intraoperative data that exceed manual processing capabilities, AI will play an indispensable role in this integration [81]. AI can analyze complex, real-time datasets; identify patterns; and provide surgeons with actionable, evidence-based recommendations, significantly reducing reliance on subjective assessment and trial-and-error adjustments. Early applications of AI in TKA have shown promise in predicting optimal surgical strategies and outcomes by leveraging historical surgical data and real-time intraoperative measurements [82–84].

Nevertheless, several fundamental challenges still exist, including establishing clear definitions of a “truly balanced knee” and “soft-tissue balance”, and identifying optimal strategies to effectively achieve a the delicate balance of the Gap–Alignment–Force triad during TKA. Addressing these conceptual and theoretical issues is essential for guiding and optimizing the effective application of digital tensioners in TKA.

## Limitations

This review has several limitations. First, although we attempted a comprehensive literature search, it is possible that some relevant articles or tensioning devices were not included, especially newer technologies under development or older devices that were not well-documented in the literature. Second, owing to scope and space constraints, we focused primarily on devices that have been clinically adopted and on well-documented devices; there exist numerous proprietary or experimental tensor designs, particularly from the mechanical tensor era, that were not detailed in this review [84–87]. Third, while we comprehensively presented product features, design principles, advantages, limitations, and historical development of tensioning devices, we could not fully elaborate on their practical limitations in surgical settings, such as operational complexity, price issues, technical training requirements, and learning curves, as these aspects are infrequently reported in the literature. Fourth, clinical evidence in this field is evolving—the outcome data for digital tensioners, especially active systems, are mostly short term. Long-term outcome data from larger cohorts are still needed to fully assess the impact on implant longevity and patient satisfaction. Fifth, while most studies reviewed reported the inclusion of both male and female patients, they rarely provided detailed sex-specific subgroup analyses. The absence of these analyses limits our ability to determine potential sex-related differences in outcomes, which may affect the generalizability of our findings. Finally, advanced digital and robotic devices typically have high costs and limited availability, especially in low-resource clinical settings, potentially restricting their widespread use. Furthermore, the associated learning curve and required training time for surgeons may delay the clinical adoption and implementation of these sophisticated technologies. Additionally, soft-tissue balancing represents only one aspect contributing to the overall success of TKA; implant design differences, patient-specific alignment philosophies, and rehabilitation protocols also significantly influence outcomes. This review focused largely on the devices and their direct effects, and other factors were assumed to be constant. However, we acknowledge that this assumption may not always hold true in clinical practice, representing an important limitation of our review. Nevertheless, we believe that, by synthesizing historical developments with contemporary evidence, this review provides a useful framework and identifies important considerations for both current practice and future investigations of soft-tissue balancing in TKA.

## Conclusions

Over the past five decades, soft-tissue tension gauging methods have evolved from experiential techniques involving mechanical devices to sophisticated digital and analytical tensor technologies, significantly enhancing our understanding of knee balance and intraoperative consistency. Current clinical evidence suggests modest improvements in patient outcomes with active digital tensors. The integration of digital tensioners with robotic systems has emerged as the most promising advancement. This integration enables precise quantification and real-time adjustment of soft-tissue tension forces, joint gaps, and limb alignment, thereby supporting improved surgical decision-making. Managing the complexity and extensive intraoperative data generated by these integrated systems necessitates advanced AI solutions capable of interpreting and converting data into actionable surgical guidance. Consequently, the continued development of AI-driven data processing and integration technologies represents the next critical frontier in refining soft-tissue balancing strategies. Ultimately, these technological advancements hold substantial clinical potential to further improve patient satisfaction, reduce complications, optimize functional outcomes, and enhance overall clinical efficacy in TKA.

## Data Availability

The datasets used and analyzed during the study will be available from the corresponding authors upon reasonable request.

## Abbreviations

TKA: Total knee arthroplasty
AI: Artificial intelligence
ROM: Range of motion
PRISMA: Preferred Reporting Items for Systematic Reviews and Meta-analyses
PROSPERO: International Prospective Register of Systematic Reviews
OLED: Organic light-emitting diode
DKBS: Dynamic Knee Balancing System
UKA: Unicompartmental knee arthroplasty
GAF: Gap–Alignment–Force
PROM: Patient-reported outcome measures
KSS: Knee Society Score
WOMAC: Western Ontario and McMaster Universities Osteoarthritis Index
